# Predictive model and scoring system for delayed cerebral ischemia following aneurysmal subarachnoid hemorrhage: A ten-year prospective analysis of observational data

**DOI:** 10.1016/j.bas.2025.105885

**Published:** 2025-11-19

**Authors:** Djula Djilvesi, Dragan Nikolic, Marijana Basta Nikolic, Bojan Jelaca, Jagos Golubovic, Filip Pajicic, Nebojsa Lasica

**Affiliations:** a Clinic of Neurosurgery, University Clinical Center of Vojvodina, Novi Sad, Serbia; b Faculty of Medicine, University of Novi Sad, Novi Sad, Serbia; c University Clinical Center of Vojvodina, Clinic for Vascular and Endovascular Surgery, Novi Sad, Serbia; d Center for Radiology, University Clinical Center of Vojvodina, Novi Sad, Serbia

**Keywords:** Aneurysmal subarachnoid hemorrhage, Delayed cerebral ischemia, Cerebral vasospasm, Predictive model, Risk stratification, Scoring system

## Abstract

**Introduction:**

Cerebral vasospasm after aneurysmal subarachnoid hemorrhage (aSAH) may cause delayed cerebral ischemia (DCI), a major determinant of poor outcomes. Existing predictive models lack dynamic clinical assessment, limiting timely intervention.

**Research question:**

This study aimed to develop and validate a model integrating baseline and dynamic predictors of DCI.

**Materials and methods:**

We analyzed data from 524 aSAH patients admitted between 2015 and 2024. Multivariate logistic regression identified predictors of DCI, which were incorporated into the Delayed Cerebral Ischemia Scoring System (DCISS). Model performance was internally validated.

**Results:**

DCI occurred in 157 patients (30 %). Seven independent predictors were identified: age <55 years (aOR 2.24), modified Fisher grades 3–4 (aOR 2.92), pathological blood pressure (aOR 1.76), pathological body temperature (aOR 2.38), restlessness (aOR 2.10), new neurological deficit (aOR 4.85), and Glasgow Coma Scale deterioration ≥2 points (aOR 6.33). The DCISS demonstrated excellent discrimination (AUC .89, 95 % CI .86–.92) with 88.5 % sensitivity and 82.0 % specificity at a cut-off ≥8. Risk groups were defined: low (0–3 points, 3.2 % risk), moderate (4–7, 18.5 %), high (8–13, 46.7 %), and very high (≥14, 82.5 %). Higher scores correlated with unfavorable outcomes (mRS 3–6) at discharge and 3 months (p < .001).

**Discussion and conclusion:**

The DCISS, incorporating static and dynamic parameters, offers robust prediction of DCI after aSAH. By enabling continuous risk re-stratification and individualized management, it may improve early identification of high-risk patients and reduce morbidity.

## Highlights

• Ten-year, single-center aSAH cohort.
• New DCISS score predicts delayed cerebral ischemia.
• DCISS shows high discrimination (AUC .89).
• Facilitates early risk stratification and intervention.

## 1 Introduction

Aneurysmal subarachnoid hemorrhage (aSAH) remains one of the most devastating cerebrovascular emergencies, affecting approximately 9 per 100,000 person-years globally (Etminan et al., 2019; Lawton and Vates, 2017). This condition carries substantial mortality rates of 25–35 % and leaves approximately one-third of survivors with significant functional impairment (Nieuwkamp et al., 2009; van et al., 2007). The high morbidity associated with aSAH is largely attributable to delayed complications that develop following the initial hemorrhage, with delayed cerebral ischemia (DCI) representing one of the most clinically significant (Dority and Oldham, 2016).

Vasospasm occurs in 60–70 % of patients with aSAH, typically developing between days 3–14 after the initial bleeding event (Macdonald and Schweizer, 2017; Budohoski et al., 2014). While radiological evidence of vasospasm can be detected in almost all patients (Djilvesi et al., 2020a), DCI occurs in approximately 20–30 % of cases (Vergouwen et al., 2010a; Shirao et al., 2011). In line with current consensus definitions, vasospasm refers to the radiological finding of arterial narrowing, whereas DCI indicates the clinical deterioration characterized by new neurological deficits or cerebral infarction not attributable to other causes.

The pathogenesis of vasospasm is initiated by blood breakdown products in the subarachnoid space. Oxyhemoglobin released triggers calcium-dependent and calcium-independent contractile mechanisms within vascular smooth muscle cells, while simultaneously decreasing nitric oxide bioavailability and increasing endothelin-1 production (Pluta et al., 2009; Macdonald et al., 2007a). This vasoconstrictive cascade is further exacerbated by inflammatory processes, oxidative stress, and microthrombosis, ultimately leading to reduced cerebral perfusion and potential ischemic injury (Carr et al., 2013; Kamp et al., 2014).

Early identification of patients at high risk for developing DCI would potentially provide critical window for intervention. Several risk factors have been previously investigated, including the volume of subarachnoid blood on initial imaging, patient age, neurological status at admission, intracranial aneurysm location, and the presence of intraventricular hemorrhage (Inagawa et al., 2014; Shibuya et al., 1992; Pickard et al., 1989). However, existing predictive models predominantly focus on static admission parameters, failing to incorporate the dynamic clinical changes that may foreshadow impending DCI (Treggiari et al., 2003a; Al- et al., 2010).

The most widely used predictive tool, the modified Fisher scale has demonstrated utility in predicting DCI risk (Claassen et al., 2001). However, it does factor in the evolving clinical parameters that may arise during the vulnerable post-ictal period. More comprehensive models have been proposed, but most lack validation in large patient cohort or omit including longitudinal clinical assessment (Wilson et al., 2012; Dumont et al., 2003).

Previous research from our group has demonstrated that angiographic vasospasm (AVS) is a common phenomenon following aSAH, with varying degrees and extent that do not readily correlate with clinical manifestations (Djilvesi et al., 2020a). Specific clinical signs and symptoms, including arterial blood pressure, elevated body temperature, restlessness, and neurological deterioration, have also been identified as strongly associated with the development of DCI (Doerksen and Naimark, 2006a). These findings suggest that a more comprehensive approach to DCI prediction—integrating baseline risk factors and evolving clinical parameters—may improve risk stratification.

Recent advances in understanding the complex pathophysiology of vasospasm have highlighted potential new strategies for DCI prediction. Metabolomic profiling has identified several potential biomarkers associated with vasospasm development, including alterations in amino acid metabolism, energy substrates, and inflammatory mediators (Lasica et al., 2023; Penn et al., 2014). Furthermore, novel neuroimaging techniques such as CT perfusion and diffusion-weighted MRI have showed promising results in detecting early perfusion abnormalities prior to evident clinical deterioration (Wintermark et al., 2006; Killeen et al., 2011).

This study aimed to investigate a novel prediction model incorporating both baseline and dynamic clinical parameters from a 10-year single-center patient cohort to develop risk stratification that would provide therapeutic window for preventive measures before irreversible neurological damage occurs. By bridging the gap between static and dynamic risk factors, the validated scoring system aims to provide a practical bedside tool for early DCI detection, addressing the multifactorial pathophysiology of vasospasm and optimizing outcomes in this vulnerable population.

## 2 Materials and methods

This study is a retrospective analysis of prospectively collected observational data from patients with aSAH admitted to the Clinic of Neurosurgery, University Clinical Center of Vojvodina, Novi Sad, Serbia, between January 2015 and December 2024. This single-center study was conducted in accordance with the Declaration of Helsinki and received approval from the Institutional Review Board (Ethics approval number: 00–20/118-2015).

The electronic medical records and neuroimaging databases were queried to include all adult patients with non-traumatic SAH. The diagnosis of aSAH was established based on non-contrast computed tomography (CT) findings of subarachnoid blood or by the presence of blood in cerebrospinal fluid (CSF) obtained with lumbar puncture, and identification of an intracranial aneurysm (IA) as the bleeding source using CT angiography (CTA) and/or digital subtraction angiography (DSA).

### 2.1 Study design and inclusion criteria

Patients were eligible for inclusion if they met the following criteria: (1) 18 years or older; (2) radiologically confirmed aSAH with identifiable IA; (3) admission to our institution within 72 h of ictus onset; (4) documented time of ictus or at least reasonably estimated time of ictus onset; and (5) complete medical records with sufficient clinical and radiological data for analysis.

Exclusion criteria were: (1) non-aneurysmal SAH (traumatic, arteriovenous malformation-related, etc.) (2) AVS evidence at initial presentation; (3) prior treatment for IA; (4) significant procedural complications during IA treatment (e.g., intraoperative rupture with significant hemorrhage, parent vessel occlusion, or large territorial infarct); (5) severe; comorbidities that could confound the assessment of DCI-related symptoms (e.g., end-stage renal disease, decompensated heart failure, or active malignancy); and (6) death within 72 h of admission unrelated to DCI.

### 2.2 Data collection and variable assessment

Data were retrospectively extracted from electronic medical records using a standardized collection form, consistent with a retrospective chart review design. Two independent investigators (D.D. and M.B.N.) performed the data extraction, with a third investigator (B.J.) resolving any discrepancies. Variables were classified into four categories: demographic characteristics, admission parameters, treatment variables, and outcome measures.

### 2.3 Demographic and baseline clinical data

Patient age, sex, medical history (including arterial hypertension, diabetes mellitus, and history of tobacco use), and pre-existing neurological conditions were recorded. The time from symptom onset to hospital admission was documented to the nearest hour based on witness accounts or emergency medical service records.

Initial clinical severity of aSAH was assessed using the World Federation of Neurosurgical Societies (WFNS) scale, with patients dichotomized into two categories: good-grade (WFNS grades I-III) or poor-grade (WFNS grades IV-V). Level of consciousness was assessed using the Glasgow Coma Scale (GCS), and baseline focal neurological deficits were systematically documented in medical records. Laboratory parameters—complete blood count, electrolytes, renal function, coagulation profile, and inflammatory markers—were obtained from admission blood samples.

### 2.4 Radiological assessment

All patients underwent standardized neuroimaging protocol at admission. The extent of subarachnoid blood was graded according to the original Fisher scale (Fisher et al., 1980a) and the modified Fisher scale (Claassen et al., 2001). Additional hemorrhagic complications including intraventricular hemorrhage, intracerebral hematoma, and subdural hemorrhage were documented, as was the presence of acute hydrocephalus. CTA was performed following non-contrast CT using a standardized dual-energy protocol.

IA characteristics including location (anterior versus posterior circulation), size (maximum diameter in millimeters), morphology (saccular, fusiform, or dissecting), and multiplicity were assessed by two independent board-certified neuroradiologists with two years of experience blinded to clinical outcomes.

### 2.5 Treatment strategy and management protocol

All patients were managed according to a standardized institutional protocol that was based on international guidelines (Connolly et al., 2012).

### 2.6 Clinical monitoring and DCI surveillance

The following parameters were systematically assessed and documented:

1. Arterial blood pressure was measured hourly in the intensive care unit and every 2 h on the ward. Pathological values were defined as systolic pressure >160 mmHg or <90 mmHg, or diastolic pressure >90 mmHg or <60 mmHg.
2. Body temperature was recorded every 2 h, with pathological values defined as temperature >37.5 °C.
3. Presence of headache was assessed using a standardized 11-point numerical rating scale (0–10) every 2 h in conscious patients. Significant headache was defined as new-onset or worsening headache (increase ≥2 points from baseline) that was not attributable to other causes.
4. Restlessness was documented by nursing staff every 2 h and defined as new-onset or increased agitation, anxiety, or non-purposeful movements not attributable to pain, delirium, or other systemic causes.
5. Comprehensive neurological examinations were performed by neurosurgical residents or attending physicians every 6 h, with additional examinations if any concerning changes were noted by nursing staff. These examinations systematically assessed: level of consciousness (using the GCS score), with separate scoring for right- and left-sided motor response, pupillary size and reactivity, speech and language function, motor strength in all extremities, sensory function, cranial nerve function, and cerebellar function when testable.

New neurological deficit was defined as any new focal neurological finding (e.g., hemiparesis, aphasia, cranial nerve palsy) not present on admission and not attributable to other causes such as seizures, hydrocephalus, rebleeding, metabolic derangements, or infection (Schmidt et al., 2008). Significant neurological deterioration was defined as a decrease in the GCS by ≥ 2 points compared to the best neurological status post-IA treatment.

### 2.7 AVS detection and classification

All patients underwent CTA at admission (baseline) and follow-up CTA 9 days post-ictus or earlier if clinical decline was noted and with a presumed diagnosis of DCI. Cerebral blood vessel diameters were measured at standardized locations on admission and follow-up CTA using Syngo.via CT Vascular Analysis software (Siemens Healthcare). Assessed segments included supraclinoid internal carotid artery (ICA), M1 segment of middle cerebral artery (MCA), A1/A2 segments of anterior cerebral artery (ACA), P1 segment of posterior cerebral artery (PCA), vertebral arteries (VA), and basilar artery (BA).

AVS was defined as arterial narrowing compared to baseline CTA at admission and categorized by severity as following: mild (5–33 % diameter reduction), moderate (34–66 %), or severe (67–100 %) AVS. Distribution was classified as local (single unilateral territory), regional (multiple unilateral), or global (bilateral multiple territories).

DCI was defined according to criteria defined by Vergouwen et al. In brief: (1) neurological decline (new deficit or GCS decline ≥2 points) not attributable to re-bleeding, hydrocephalus, seizure, metabolic disturbances, sedation, or infection; and (2) new infarction identified on CT or MRI, or proven at autopsy, within 6 weeks after SAH (or on the last scan before death within that window); should not have been present on a CT/MRI done 24–48 h post aneurysm occlusion (to exclude procedural infarctions) or clearly attributable to other causes (e.g., hemorrhage, surgical trauma, catheter‐induced, etc.) (Frontera et al., 2009; Vergouwen et al., 2010b).

### 2.8 Outcome assessment

Primary outcome: DCI during hospitalization.

Secondary outcomes:

1. Functional outcome (hospital discharge/3-month post-ictus) assessed via modified Rankin Scale (favorable mRS 0–2).
2. Intensive care unit (ICU) and total hospital stay duration.
3. In-hospital mortality.

### 2.9 Statistical analysis

Based on the primary aim of developing a predictive model for DCI, a minimum of 150 DCI events (10 events per predictor variable for 15 variables) was required. Assuming a 30 % DCI incidence rate from prior studies (Vergouwen et al., 2010a), this necessitated a sample size of ≥500 patients, that was exceeded with the final cohort of 524 patients. Statistical analysis was performed using SPSS version 27.0 (IBM Corp., Armonk, NY, USA) and R version 4.1.2 (R Foundation for Statistical Computing, Vienna, Austria). Continuous variables were reported as means with standard deviations or medians with interquartile ranges, depending on distribution normality as assessed by the Shapiro-Wilk test. Categorical variables were presented as absolute numbers and percentages.

Comparisons between patients who developed DCI and those who did not were performed using the independent samples *t*-test or Mann-Whitney *U* test for continuous variables and Pearson's chi-square test or Fisher's exact test for categorical variables, as appropriate. For all analyses, a two-tailed p-value <.05 was considered statistically significant.

Univariate logistic regression analysis was conducted to identify factors associated with DCI development. Variables with a p-value <.10 in univariate analysis were considered for inclusion in the multivariate model. Multicollinearity was assessed using variance inflation factor (VIF) analysis, with values > 5 indicating significant multicollinearity.

### 2.10 Multivariate analysis and predictive model development

Multivariate logistic regression with backward stepwise elimination (likelihood ratio method) was used to identify independent predictors of DCI. Variables were removed from the model if they did not contribute significantly (p > .05) to the model's predictive ability. The final model included only statistically significant independent predictors.

The predictive model's performance was assessed using the area under the receiver operating characteristic curve (AUC-ROC), with 95 % confidence intervals calculated using the DeLong method. Calibration was evaluated using the Hosmer-Lemeshow goodness-of-fit test and visual inspection of calibration plots.

### 2.11 Scoring system development and validation

A clinical scoring system was developed based on the regression coefficients from the final multivariate model. Each independent predictor was assigned a weighted point value proportional to its regression coefficient, with the predictor having the smallest statistically significant coefficient assigned 1 point and other predictors assigned points relative to this reference.

The optimal cut-off score for predicting high risk of DCI was determined using the Youden index (maximizing the sum of sensitivity and specificity). Sensitivity, specificity, positive predictive value, negative predictive value, and accuracy were calculated for the chosen cut-off.

Internal validation of the prediction model was performed using bootstrapping with 1000 resamples to quantify optimism in the model's predictive performance and to provide bias-corrected estimates of discrimination and calibration. The model's clinical utility was assessed using decision curve analysis, which quantifies the net benefit of using the model across a range of threshold probabilities.

Risk categories were established based on the score distribution and corresponding risk of DCI: low risk (<5 % probability), moderate risk (5–30 % probability), high risk (30–60 % probability), and very high risk (>60 % probability).

## 3 Results

### 3.1 Patient population and baseline characteristics

A total of 524 patients with an aneurysmal subarachnoid hemorrhage (aSAH) met the inclusion criteria and were enrolled in the study. The cohort consisted of 304 females (58.0 %) and 220 males (42.0 %), with a mean age of 54.3 ± 10.8 years (range: 18–75 years). Most patients (n = 271, 51.7 %) were younger than 55 years of age. Baseline demographic and clinical characteristics of the study population are presented in Table 1.

**Table 1:** Demographic and Clinical Characteristics of Patients with aSAH.

Characteristics	Total Cohort (n = 524)	DCI (n = 157)	No DCI (n = 367)	p-value
Demographics				
Age (years), mean ± SD	54.3 ± 10.8	49.6 ± 11.3	56.4 ± 9.9	<.001∗
Age <55 years, n (%)	271 (51.7)	108 (68.8)	163 (44.4)	<.001∗
Female sex, n (%)	304 (58.0)	98 (62.4)	206 (56.1)	.182
Clinical Presentation				
Ictus to admission (hours), median (IQR)	14.5 (6.2–28.3)	12.8 (5.6–26.1)	15.2 (6.7–29.2)	.203
Admission within 24 h, n (%)	347 (66.2)	109 (69.4)	238 (64.9)	.315
WFNS grade I, n (%)	192 (36.6)	45 (28.7)	147 (40.1)	–
WFNS grade II, n (%)	142 (27.1)	46 (29.3)	96 (26.2)	–
WFNS grade III, n (%)	98 (18.7)	36 (22.9)	62 (16.9)	–
WFNS grade IV, n (%)	69 (13.2)	23 (14.6)	46 (12.5)	–
WFNS grade V, n (%)	23 (4.4)	7 (4.5)	16 (4.4)	.032∗
Poor WFNS grade (III-V), n (%)	190 (36.3)	66 (42.0)	124 (33.8)	.073
Glasgow Coma Scale, mean ± SD	13.2 ± 2.9	12.7 ± 3.1	13.4 ± 2.8	.015∗
Comorbidities				
Hypertension, n (%)	285 (54.4)	87 (55.4)	198 (54.0)	.762
Diabetes mellitus, n (%)	76 (14.5)	19 (12.1)	57 (15.5)	.307
Dyslipidemia, n (%)	142 (27.1)	37 (23.6)	105 (28.6)	.236
Smoking, n (%)	237 (45.2)	84 (53.5)	153 (41.7)	.012∗
Previous stroke, n (%)	32 (6.1)	8 (5.1)	24 (6.5)	.527
Radiological Findings				
Fisher grade II, n (%)	126 (24.0)	21 (13.4)	105 (28.6)	<.001∗
Fisher grade III, n (%)	178 (34.0)	61 (38.9)	117 (31.9)	–
Fisher grade IV, n (%)	220 (42.0)	75 (47.8)	145 (39.5)	–
Fisher grades III-IV, n (%)	398 (76.0)	136 (86.6)	262 (71.4)	<.001∗
Intraventricular hemorrhage, n (%)	226 (43.1)	78 (49.7)	148 (40.3)	.048∗
Intracerebral hematoma, n (%)	143 (27.3)	48 (30.6)	95 (25.9)	.267
Acute hydrocephalus, n (%)	97 (18.5)	36 (22.9)	61 (16.6)	.086
Clinical Monitoring Parameters				
Pathological arterial blood pressure, n (%)	352 (67.2)	123 (78.3)	229 (62.4)	<.001∗
Pathological body temperature, n (%)	343 (65.5)	127 (80.9)	216 (58.9)	<.001∗
Restlessness, n (%)	205 (39.1)	85 (54.1)	120 (32.7)	<.001∗
New neurological deficit, n (%)	143 (27.3)	83 (52.9)	60 (16.3)	<.001∗
Deterioration of GCS ≥2 points, n (%)	137 (26.1)	87 (55.4)	50 (13.6)	<.001∗

**Abbreviations:** SD = standard deviation; IQR = interquartile range; WFNS = World Federation of Neurosurgical Societies; GCS = Glasgow Coma Scale.

**Note:** Pathological arterial blood pressure was defined as systolic pressure >160 mmHg or <90 mmHg, or diastolic pressure >90 mmHg or <60 mmHg. Pathological body temperature was defined as temperature >37.5 °C.

∗ Statistically significant (p < .05).

Most patients (n = 434, 82.8 %) were admitted within 48 h of symptom onset, with a median time from ictus to admission of 14.5 h (IQR: 6.2–28.3). At presentation, WFNS grades were distributed as follows: grade I (192 patients, 36.6 %), grade II (142, 27.1 %), grade III (98, 18.7 %), grade IV (69, 13.2 %), and grade V (23, 4.4 %). The mean GCS score on admission was 13.2 ± 2.9.

Arterial hypertension (n = 285, 54.4 %), history of tobacco use (n = 237, 45.2 %), and dyslipidemia (n = 142, 27.1 %) were the most common comorbidities. A minority of patients (n = 53, 10.1 %) had pre-existing neurological conditions, including prior stroke (n = 32, 6.1 %), and seizure (n = 11, 2.1 %).

### 3.2 Radiological findings and aneurysm characteristics

Initial CT findings classified by the Fisher scale revealed grade II hemorrhage (n = 126, 24.0 %), grade III (n = 178, 34.0 %), and grade IV (n = 220, 42.0 %). Using the modified Fisher scale, patients were distributed as grade I (n = 103, 19.7 %), grade II (n = 89, 17.0 %), grade III (n = 152, 29.0 %), and grade IV (n = 180, 34.3 %). Complications included intraventricular hemorrhage (n = 226, 43.1 %), intracerebral hematoma (n = 143, 27.3 %), and acute hydrocephalus requiring CSF diversion (n = 97, 18.5 %). IA characteristics based on CTA and DSA findings are summarized in Table 2.

**Table 2:** Characteristics of ruptured IAs.

Characteristics	Total Cohort (n = 524)	DCI (n = 157)	No DCI (n = 367)	p-value
Aneurysm Location				
Anterior circulation, n (%)	420 (80.2)	128 (81.5)	292 (79.6)	.605
ACoA	189 (36.1)	64 (40.8)	125 (34.1)	.142
MCA	136 (26.0)	39 (24.8)	97 (26.4)	.700
ICA	73 (13.9)	21 (13.4)	52 (14.2)	.809
ACA (A2-A5)	22 (4.2)	4 (2.5)	18 (4.9)	.211
Posterior circulation, n (%)	104 (19.8)	29 (18.5)	75 (20.4)	.605
PCoA	63 (12.0)	18 (11.5)	45 (12.3)	.800
BA	42 (8.0)	11 (7.0)	31 (8.4)	.582
VA	14 (2.7)	3 (1.9)	11 (3.0)	.477
PICA	10 (1.9)	2 (1.3)	8 (2.2)	.490
Multiple aneurysms, n (%)	87 (16.6)	24 (15.3)	63 (17.2)	.593
Aneurysm Size				
Aneurysm size (mm), median (IQR)	6.8 (4.5–9.2)	6.5 (4.3–9.0)	6.9 (4.6–9.3)	.427
Size category, n (%)				.651
Small (<7 mm)	306 (58.4)	96 (61.1)	210 (57.2)	–
Medium (7–12 mm)	172 (32.8)	48 (30.6)	124 (33.8)	–
Large (13–24 mm)	39 (7.4)	11 (7.0)	28 (7.6)	–
Giant (≥25 mm)	7 (1.3)	2 (1.3)	5 (1.4)	–
Aneurysm Morphology				
Morphology, n (%)				.873
Saccular	463 (88.4)	138 (87.9)	325 (88.6)	–
Fusiform	42 (8.0)	13 (8.3)	29 (7.9)	–
Dissecting	19 (3.6)	6 (3.8)	13 (3.5)	–
Dome-to-neck ratio >2, n (%)	217 (41.4)	68 (43.3)	149 (40.6)	.562
Irregular shape/daughter sac, n (%)	178 (34.0)	59 (37.6)	119 (32.4)	.252
Treatment Variables				
Treatment modality, n (%)				.312
Microsurgical clipping	241 (46.0)	78 (49.7)	163 (44.4)	–
Endovascular coiling	210 (40.1)	57 (36.3)	153 (41.7)	–
Conservative management	73 (13.9)	22 (14.0)	51 (13.9)	–
Ictus to treatment (h), median (IQR)	28.3 (18.5–64.7)	26.1 (17.2–58.4)	29.5 (19.3–67.8)	.183
Early treatment (≤72h), n (%)∗	395 (87.6)	119 (88.1)	276 (87.3)	.814
Procedural complications, n (%)∗	42 (9.3)	13 (9.6)	29 (9.2)	.881

**Abbreviations:** IQR = interquartile range; ACoA = Anterior Communicating Artery; MCA = Middle Cerebral Artery; ICA = Internal Carotid Artery; ACA = Anterior Cerebral Artery; PcoA = Posterior Communicating Artery; BA = Basilar Artery; VA = Vertebral Artery; PICA = Posterior Inferior Cerebellar Artery.

**Note:** ∗Percentages for early treatment and procedural complications are calculated based on the number of treated patients (n = 451) rather than the total cohort.

No statistically significant differences were observed in aneurysm characteristics between patients who developed delayed cerebral ischemia (DCI) and those who did not.

Most IAs were located in the anterior circulation (n = 420, 80.2 %), with the most common sites being the ACoA (n = 189, 36.1 %), MCA (n = 136, 26.0 %), and ICA (73, 13.9 %). Posterior circulation aneurysms accounted for 104 cases (19.8 %), predominantly involving the BA (n = 42, 8.0 %) and PcoA (n = 63, 12.0 %). Multiple intracranial aneurysms were identified in 87 patients (16.6 %).

The median maximum diameter of the ruptured IAs was 6.8 mm (IQR: 4.5–9.2 mm), with 306 IAs (58.4 %) classified as small (<7 mm), 172 (32.8 %) as medium (7–12 mm), 39 (7.4 %) as large (13–24 mm), and 7 (1.3 %) as giant (≥25 mm). Regarding morphology, 463 IAs (88.4 %) were saccular, 42 (8.0 %) were fusiform, and 19 (3.6 %) were dissecting.

Of the 524 patients, 451 (86.1 %) underwent IA treatment, with 241 patients (46.0 %) treated microsurgically and 210 (40.1 %) treated endovascularly. The remaining 73 patients (13.9 %) were managed conservatively due to poor clinical condition (29 patients, 5.5 %), unfavorable aneurysm configuration (32 patients, 6.1 %), or patient/family preference (12 patients, 2.3 %). In the endovascular group, 183 patients (87.1 %) were treated with standard coiling, 21 (10.0 %) with stent-assisted coiling, and 6 (2.9 %) with flow diversion devices.

The median time from ictus to treatment was 28.3 h (IQR: 18.5–64.7). Early treatment (within 72 h of ictus) was achieved in 395 patients (87.6 % of those treated). Procedure-related complications occurred in 42 patients (9.3 %), including intraoperative rupture in 18 patients (4.0 %), embolic events in 15 patients (3.3 %), and parent vessel occlusion in 9 patients (2.0 %). These complications resulted in neurological worsening in 23 patients (5.1 %).

### 3.3 Incidence and characteristics of AVS

**Fig. 1:**
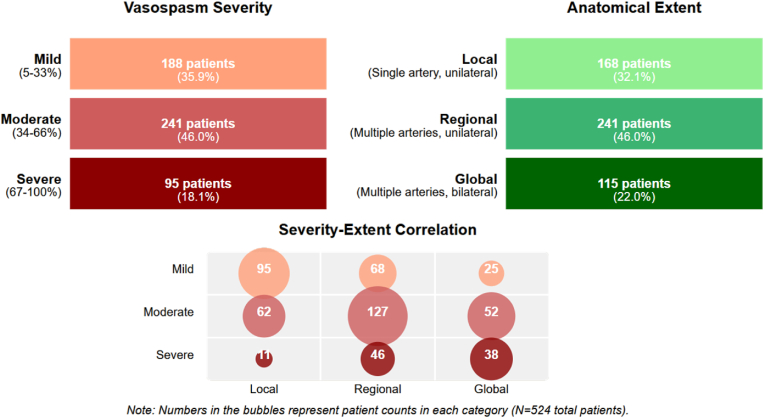
Distribution of AVS by Severity and Anatomical Extent. Figure illustrates AVS severity (*upper left*), the anatomical distribution (*upper right*), and the correlation between severity and anatomical extent (*bottom*), with the most common pattern being moderate-regional (n = 127 patients), followed by mild-local (n = 95 patients) and moderate-global (n = 52 patients) AVS patterns.

AVS was detected on follow-up CTA in all 524 patients, according to the aforementioned criteria, with varying extent and degree of severity. The distribution of AVS is illustrated in Fig. 1.

This finding suggests that AVS was an universal phenomenon—a notably higher detection rate than typically reported—and underscores the importance of distinguishing radiological vasospasm from clinically manifest delayed cerebral ischemia (DCI).

**Fig. 2:**
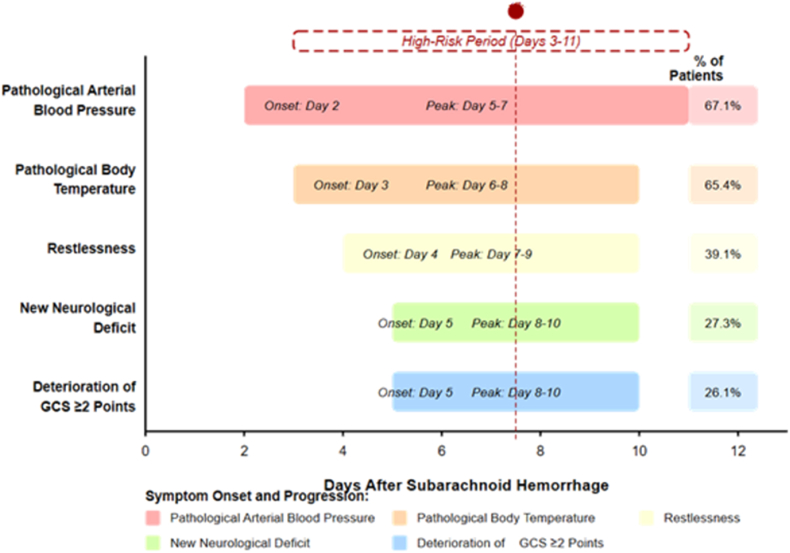
Time Course of Clinical Symptoms and Signs Associated with AVS Development. Horizontal bars depict the onset and duration of each manifestation, with specific days and peak periods annotated. Pathological arterial blood pressure (67.1 % of patients) first appeared on day 2, peaking between days 5–7. Pathological body temperature (65.4 %) followed, with onset around day 3 and peak incidence at days 6–8. Restlessness (39.1 %) typically began on day 4 and peaked at days 7–9. New neurological deficits (27.3 %) and deterioration of the GCS (≥2 points; 26.1 %) emerged later, starting on day 5 and peaking between days 8–10, which coincided with the maximal incidence of AVS. The highlighted area marks the high-risk period (days 3–11), when intensive monitoring is critical for early detection and intervention.

The median time from ictus to AVS detection was 7.2 days (IQR: 5.4–9.6). AVS onset occurred before day 4 in 19 patients (3.6 %), during days 4–6 in 152 patients (29.0 %), during days 7–10 in 289 patients (55.2 %), and after day 10 in 64 patients (12.2 %). The temporal relationship between clinical symptoms/signs and AVS development is illustrated in Fig. 2.

DCI developed in 157 patients (30.0 %). Among these, 131 patients (83.4 %) had moderate or severe, while 26 (16.6 %) had mild AVS. The clinical manifestations of DCI included new focal neurological deficits in 103 patients (65.6 %), decreased level of consciousness in 96 patients (61.1 %), or both in 42 patients (26.8 %).

Cerebral infarction was evident on follow-up neuroimaging in 189 patients (36.1 %). The distribution of infarct territories is shown in Table 3.

**Table 3:** Distribution of cerebral infarction territories.

Characteristics	Total (n = 189)	DCI (n = 143)	Non-DCI (n = 46)	p-value
Arterial Territories				
MCA territory, n (%)	97 (51.3)	79 (55.2)	18 (39.1)	.046∗
Superficial (cortical)	48 (25.4)	37 (25.9)	11 (23.9)	.790
Deep (basal ganglia/internal capsule)	27 (14.3)	22 (15.4)	5 (10.9)	.440
Both superficial and deep	22 (11.6)	20 (14.0)	2 (4.3)	.074
ACA territory, n (%)	53 (28.0)	44 (30.8)	9 (19.6)	.143
Pericallosal territory	29 (15.3)	24 (16.8)	5 (10.9)	.333
Callosomarginal territory	24 (12.7)	20 (14.0)	4 (8.7)	.340
PCA territory, n (%)	19 (10.1)	12 (8.4)	7 (15.2)	.177
Occipital lobe	13 (6.9)	8 (5.6)	5 (10.9)	.210
Thalamus	6 (3.2)	4 (2.8)	2 (4.3)	.606
PICA territory, n (%)	12 (6.3)	6 (4.2)	6 (13.0)	.030∗
AICA territory, n (%)	5 (2.6)	2 (1.4)	3 (6.5)	.065
SCA territory, n (%)	3 (1.6)	0 (.0)	3 (6.5)	.011∗
Infarct Patterns				
Single vascular territory, n (%)	132 (69.8)	93 (65.0)	39 (84.8)	.010∗
Multiple vascular territories, n (%)	57 (30.2)	50 (35.0)	7 (15.2)	.010∗
Watershed/border zone infarcts, n (%)	43 (22.8)	38 (26.6)	5 (10.9)	.025∗
Cortical watershed	24 (12.7)	21 (14.7)	3 (6.5)	.144
Infarct Size and Timing				
Infarct volume (cm^3^), median (IQR)	14.3 (5.7–38.2)	18.6 (7.2–42.5)	8.1 (3.2–19.4)	.003∗
Ictus to infarct detection (days), median (IQR)	9.6 (7.3–12.8)	9.2 (7.1–12.3)	10.5 (8.2–14.1)	.085

**Abbreviations:** IQR = interquartile range; MCA = Middle Cerebral Artery; ACA = Anterior Cerebral Artery; PCA = Posterior Cerebral Artery; PICA = Posterior Inferior Cerebellar Artery; AICA = Anterior Inferior Cerebellar Artery; SCA = Superior Cerebellar Artery.

**Note:** Total percentage may exceed 100 % due to multiple infarct territories in some patients.

∗ Statistically significant (p < .05).

### 3.4 Predictors of DCI

#### 3.4.1 Univariate analysis

Univariate analysis comparing patients who developed DCI (n = 157) with those who did not (n = 367) is presented in Table 4.

**Table 4:** Univariate analysis of potential risk factors for DCI.

Variable	OR	95 % CI	p-value
Demographic Factors			
Age <55 years	2.57	1.76–3.76	<.001∗
Female sex	1.30	.89–1.91	.183
Smoking	1.62	1.11–2.35	.012
Hypertension	1.06	.73–1.54	.762
Clinical Presentation			
Admission within 24 h	1.23	.82–1.83	.316
WFNS grade III–V	1.93	1.32–2.83	.001∗
Glasgow Coma Scale ≤13	1.75	1.19–2.57	.004∗
Loss of consciousness at ictus	1.52	1.04–2.22	.031∗
Radiological Findings			
Fisher grade III–IV	3.24	2.12–4.95	<.001∗
Modified Fisher grade III–IV	3.02	1.98–4.61	<.001∗
Intraventricular hemorrhage	1.47	1.01–2.14	.045∗
Intracerebral hematoma	1.26	.84–1.90	.268
Acute hydrocephalus	1.49	.94–2.37	.087
Aneurysm Characteristics			
Anterior circulation aneurysm	1.13	.71–1.79	.605
Anterior communicating artery aneurysm	1.33	.91–1.96	.143
Aneurysm size ≥7 mm	.85	.58–1.24	.396
Multiple aneurysms	.87	.52–1.45	.593
Irregular aneurysm shape	1.26	.85–1.86	.253
Treatment Variables			
Microsurgical vs. endovascular treatment	1.24	.84–1.84	.285
Treatment delay >72 h	1.08	.57–2.03	.814
Procedural complications	1.05	.53–2.10	.881
Clinical Monitoring Parameters			
Pathological arterial blood pressure values	2.04	1.39–2.99	<.001∗
Pathological body temperature values	2.83	1.92–4.17	<.001∗
Restlessness	2.45	1.67–3.60	<.001∗
Deterioration of consciousness	3.31	2.22–4.94	<.001∗
New neurological deficit	5.28	3.44–8.10	<.001∗
Deterioration of GCS ≥2 points	6.91	4.48–10.66	<.001∗

**Abbreviations:** CI = confidence interval; WFNS = World Federation of Neurosurgical Societies; GCS = Glasgow Coma Scale.

**Note:** Pathological arterial blood pressure was defined as systolic pressure >160 mmHg or <90 mmHg, or diastolic pressure >90 mmHg or <60 mmHg. Pathological body temperature was defined as temperature >37.5 °C.

∗Statistically significant (p < .05).

Patients who developed DCI were significantly younger (mean age, 49.6 ± 11.3 vs. 56.4 ± 9.9 years; p < .001) and more frequently presented with poor WFNS grades (III–V) (42.0 % vs. 32.4 %; p = .032). Radiologically, higher Fisher grades (III–IV) were more prevalent in the DCI group (86.6 % vs. 70.3 %; p < .001). No significant associations were observed between DCI and sex, IA location (anterior vs. posterior circulation), size, presence of multiple intracranial aneurysms, or treatment modality (surgical vs. endovascular).

During hospitalization, patients with DCI exhibited significantly higher rates of pathological arterial blood pressure (78.3 % vs. 62.4 %; p < .001), elevated body temperature (80.9 % vs. 59.1 %; p < .001), restlessness (54.1 % vs. 32.7 %; p < .001), consciousness deterioration (58.0 % vs. 28.6 %; p < .001), new neurological deficit (52.9 % vs. 17.2 %; p < .001), and deterioration of the GCS score by ≥ 2 points (55.4 % vs. 15.5 %; p < .001).

Univariate logistic regression analysis identified the following factors to be significantly associated with DCI development: age <55 years (OR 2.57, 95 % CI 1.76–3.76, p < .001), WFNS grade III-IV (OR 1.93, 95 % CI 1.32–2.83, p < .001), Fisher grade III-IV (OR 3.24, 95 % CI 2.12–4.95, p < .001), pathological arterial blood pressure values (OR 2.04, 95 % CI 1.39–2.99, p < .001), pathological body temperature values (OR 2.83, 95 % CI 1.92–4.17, p < .001), restlessness (OR 2.45, 95 % CI 1.67–3.60, p < .001), deterioration of consciousness (OR 3.31, 95 % CI 2.22–4.94, p < .001), new neurological deficit (OR 5.28, 95 % CI 3.44–8.10, p < .001), and deterioration of GCS by ≥ 2 points (OR 6.91, 95 % CI 4.48–10.66, p < .001).

#### 3.4.2 Multivariate analysis

Multivariate logistic regression analysis was performed to identify independent predictors of DCI development. The final model is presented in Table 5.

**Table 5:** Multivariate logistic regression analysis of independent predictors for DCI.

Variable	β Coefficient	Standard Error	aOR	95 % CI	p-value	DCISS Points
Age <55 years	.806	.218	2.24	1.46–3.44	<.001	2
Fisher grade III–IV	1.071	.238	2.92	1.83–4.66	<.001	3
Pathological blood pressure values	.566	.218	1.76	1.15–2.70	.009	1
Pathological body temperature values	.867	.220	2.38	1.54–3.67	<.001	2
Restlessness	.742	.221	2.10	1.36–3.24	.001	2
New neurological deficit	1.579	.239	4.85	3.04–7.75	<.001	5
Deterioration of GCS ≥2 points	1.845	.238	6.33	3.96–10.11	<.001	6
Constant	−3.658	.308	–	–	<.001	–

**Abbreviations:** OR = odds ratio; CI = confidence interval; GCS = Glasgow Coma Scale; DCISS = Delayed Cerebral Ischemia Scoring System.

**Note:** Pathological arterial blood pressure was defined as systolic pressure >160 mmHg or <90 mmHg, or diastolic pressure >90 mmHg or <60 mmHg. Pathological body temperature was defined as temperature >37.5 °C.

**Model performance metrics:** Area under the ROC curve = .89 (95 % CI .86–.92); Hosmer-Lemeshow goodness-of-fit test: χ^2^ = 8.73, p = .366; Nagelkerke R^2^ = .47.

**DCISS points:** Points were assigned based on the β coefficients, with the reference value being the predictor with the lowest significant coefficient (pathological arterial blood pressure values, β = .566). Each predictor was assigned points proportional to its β coefficient value relative to the reference β coefficient.

Seven variables were identified as independent predictors: age <55 years (adjusted OR [aOR] 2.24, 95 % CI 1.46–3.44, p < .001), Fisher grade III-IV (aOR 2.92, 95 % CI 1.83–4.66, p < .001), pathological arterial blood pressure values (aOR 1.76, 95 % CI 1.15–2.70, p = .009), pathological body temperature values (aOR 2.38, 95 % CI 1.54–3.67, p < .001), restlessness (aOR 2.10, 95 % CI 1.36–3.24, p = .001), new neurological deficit (aOR 4.85, 95 % CI 3.04–7.75, p < .001), and deterioration of GCS by ≥ 2 points (aOR 6.33, 95 % CI 3.96–10.11, p < .001).

**Fig. 3:**
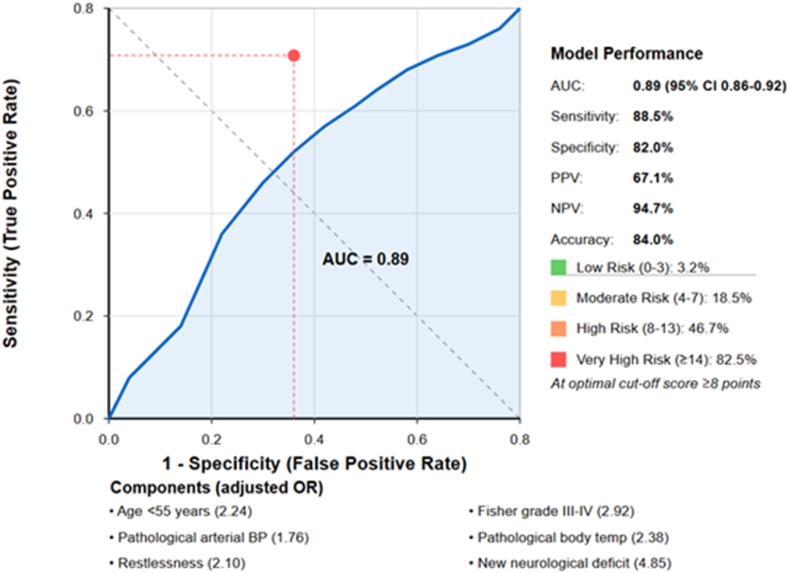
Receiver Operating Characteristic Curve for the Multivariate Predictive Model of DCI. The model demonstrates excellent discriminative ability with an area under the curve (AUC) of .89 (95 % CI .86–.92). At the optimal cut-off score of ≥8 points (indicated by the red dot), the model achieves 88.5 % sensitivity and 82.0 % specificity, with positive and negative predictive values of 67.1 % and 94.7 % respectively, resulting in an overall accuracy of 84.0 %. The Delayed Cerebral Ischemia Scoring System (DCISS) incorporates seven independent predictors with their respective adjusted odds ratios: age <55 years, Fisher grade III-IV, pathological arterial blood pressure, pathological body temperature, restlessness, new neurological deficit, and deterioration of GCS score ≥2 points. The model stratifies patients into four risk categories: low risk (0–3 points) with 3.2 % DCI risk, moderate risk (4–7 points) with 18.5 % risk, high risk (8–13 points) with 46.7 % risk, and very high risk (≥14 points) with 82.5 % risk.

The Hosmer-Lemeshow goodness-of-fit test indicated adequate model calibration (χ^2^ = 8.73, p = .366). The model demonstrated good discriminative ability with an area under the ROC curve of .89 (95 % CI .86–.92) (Fig. 3).

### 3.5 Development and validation of the Delayed Cerebral Ischemia Scoring System

Based on the aOR from the multivariate model, a a point-based DCI scoring system (DCISS) for predicting DCI was developed. Points were assigned to each independent predictor proportional to its regression coefficient, with the factor having the lowest significant aOR (pathological arterial blood pressure values, aOR 1.76) assigned 1 point. The resulting scoring system is presented in Table 6. The total DCISS score for each patient could range from 0 to 21 points.

**Table 6:** The Delayed Cerebral Ischemia Scoring System (DCISS) for Predicting DCI Following aSAH.

Risk Factor	Points
Baseline Risk Factors (assessed on admission)	
Age <55 years	2
Fisher grade III-IV (thick clot with or without intraventricular hemorrhage)	3
Dynamic Clinical Parameters (assessed during daily monitoring)	
Pathological arterial blood pressure values^a^	1
Pathological body temperature values^b^	2
Restlessness^c^	2
New neurological deficit^d^	5
Deterioration of Glasgow Coma Scale ≥2 points	6
Maximum Total Score	21

Note: The DCISS is designed to be used both on admission (baseline risk factors) and throughout the high-risk period for DCI (days 3–14), with scores updated based on changing clinical parameters. Points are cumulative, and the maximum score is 21.

**Abbreviations:** CI = confidence interval.

a Pathological arterial blood pressure values: systolic pressure >160 mmHg or <90 mmHg, or diastolic pressure >90 mmHg or <60 mmHg.

b Pathological body temperature values: temperature >37.5 °C.

c Restlessness: new-onset or increased agitation, anxiety, or non-purposeful movements not attributable to pain, delirium, or other systemic causes.

d New neurological deficit: any new focal neurological finding (e.g., hemiparesis, aphasia, cranial nerve palsy) not present on admission and not attributable to other causes such as seizures, hydrocephalus, rebleeding, metabolic derangements, or infection.

The median score in patients who developed DCI was 12 (IQR: 9–15), compared to 5 (IQR: 2–7) in those who did not (p < .001). The performance characteristics of the DCISS at various cut-off points are presented in Supplemental Digital Content 2, Table S1.

Using the Youden index, the optimal cut-off value for predicting high risk of DCI development was determined to be ≥ 8 points. At this threshold, the sensitivity was 88.5 % (95 % CI 82.5–93.1), specificity 82.0 % (95 % CI 77.7–85.8), positive predictive value 67.1 % (95 % CI 60.3–73.3), and negative predictive value 94.7 % (95 % CI 91.6–96.9). The overall accuracy was 84.0 % (95 % CI 80.5–87.0). Based on the observed risk of DCI in our cohort, we stratified patients into four risk categories: low risk: 0–3 points (observed DCI risk: 3.2 %, 95 % CI 1.3–6.5), moderate risk: 4–7 points (observed DCI risk: 18.5 %, 95 % CI 13.5–24.4), high risk: 8–13 points (observed DCI risk: 46.7 %, 95 % CI 39.2–54.3), very high risk: ≥14 points (observed DCI risk: 82.5 %, 95 % CI 72.4–90.1).

**Fig. 4:**
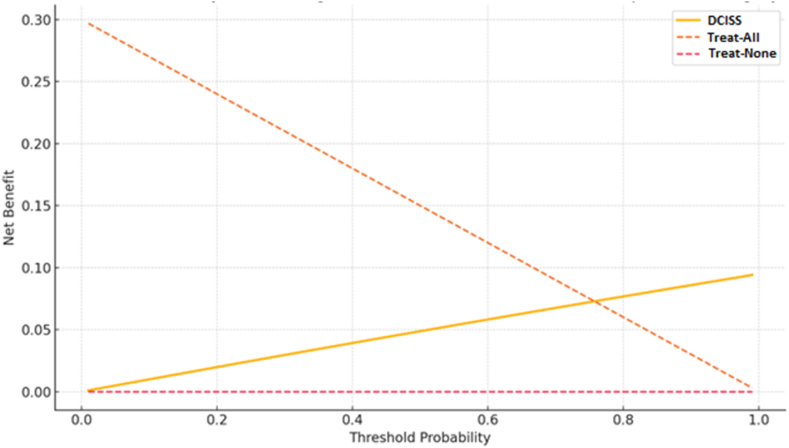
Decision Curve Analysis Showing Net Benefit of the DCISS. The graph demonstrates the clinical utility of the DCISS model across a range of threshold probabilities (x-axis) compared to alternative clinical strategies. The y-axis represents net benefit, calculated as the proportion of true positives minus the proportion of false positives weighted by the relative harm of false-positive to false-negative results. The DCISS model (solid yellow line) provides superior net benefit compared to both the “treat all” strategy (orange dashed line) and the “treat none” strategy (red dashed line) at threshold probabilities above .7, indicating its clinical value in high-stakes decision making. At lower thresholds (<.7), the “treat all” approach yields greater net benefit, suggesting that when the threshold for intervention is low, treating all patients without model-based selection may be preferable. The “treat none” strategy (net benefit of zero) serves as the reference line representing no interventions. The analysis shows that the DCISS model would add clinical value when clinicians or patients require at least a 70 % probability of DCI before initiating aggressive preventive interventions or closer monitoring. The curves were derived from bootstrap-validated predictions in the study cohort (n = 524) and demonstrate the model's robust performance across a clinically relevant range of decision thresholds. This analysis confirms that using the DCISS to guide clinical decision-making would improve patient outcomes compared to strategies that either treat all patients or treat none.

Internal validation using bootstrap resampling (1000 replicates) demonstrated minimal optimism in the model's performance, with a bias-corrected AUC of .88 (95 % CI .85–.91). The calibration slope was .93, indicating minimal overfitting. Decision curve analysis (Fig. 4) showed that the DCISS provided a positive net benefit across a wide range of threshold probabilities (10–80 %), demonstrating its clinical utility.

### 3.6 Clinical outcomes

The median length of stay in the intensive care unit was 7 days (IQR: 3–12), and the median total hospital length of stay was 18 days (IQR: 14–25). Patients who developed DCI had significantly longer ICU stays (median 12 vs. 5 days, p < .001) and total hospital stays (median 23 vs. 16 days, p < .001) compared to those without DCI.

In-hospital mortality occurred in 68 patients (13.0 %). The mortality rate was significantly higher in patients who developed DCI compared to those who did not (24.8 % vs. 8.2 %, p < .001). The causes of death are summarized in Supplemental Digital Content 2, Table S2.

Cerebral infarction was the leading cause (39.7 %), followed by direct effects of the initial hemorrhage (29.4 %), and rebleeding (13.2 %). At discharge, 291 patients (55.5 %) had favorable outcomes (mRS 0–2), while 233 (44.5 %) had unfavorable outcomes (mRS 3–6). The relationship between DCISS score categories and functional outcomes at discharge is illustrated in Supplemental Digital Content 1, Fig. S1.

At the 3-month follow-up, available for 489 patients (93.3 %), 342 patients (70.0 %) had favorable outcomes (mRS 0–2). The proportion of patients with favorable outcomes at 3 months was significantly lower in the DCI group compared to those without DCI (45.3 % vs. 81.1 %, p < .001). The distribution of mRS scores at 3 months according to DCISS risk categories is presented in Supplemental Digital Content 1, Fig. S2. This demonstrated a strong association between higher DCISS scores and worse functional outcomes.

## 4 Discussion

This 10-year analysis of aSAH patient management confirms that AVS is a universal phenomenon following IA rupture, which aligns with the prior institutional findings (Djilvesi et al., 2020b). However, DCI manifesting as neurological deterioration and potentially resulting in cerebral ischemia occurred in approximately one-third of patients (30.0 %), consistent with reported incidence rates of 20–30 % in contemporary literature (Macdonald, 2014; Diringer et al., 2011). This discrepancy between AVS and DCI suggests that the mechanisms underlying DCI pathophysiology and its relation to vasospasm are considerably more complex than previously conceptualized.

### 4.1 Risk factors and independent predictors of DCI

Seven independent predictors for DCI development: younger age (<55 years), higher Fisher grade (III-IV), pathological arterial blood pressure values, pathological body temperature values, restlessness, new neurological deficit, and deterioration on the GCS by ≥ 2 points. These findings represent a comprehensive clinical profile that encompasses both static baseline characteristics and dynamic clinical parameters.

Younger age as a risk factor for DCI has been consistently demonstrated in multiple studies (Ryttlefors et al., 2010; Malinova et al., 2016). The study cohort, patients younger than 55 years exhibited more than twice the risk for developing DCI (aOR 2.24, 95 % CI 1.46–3.44). This association may be explained by the enhanced reactivity of cerebral vessels in younger individuals and a more robust inflammatory response to blood products in the subarachnoid space (Findlay et al., 1989). Additionally, the higher prevalence of comorbidities and reduced vascular compliance in elderly patients may paradoxically attenuate vasoreactive responses to subarachnoid blood (McGirt et al., 2003).

The Fisher scale, which quantifies subarachnoid blood on initial CT imaging, proved to be a significant predictor of AVS and DCI, corroborating findings from numerous previous investigations (Fisher et al., 1980b; Frontera et al., 2006). Patients with Fisher grades III-IV demonstrated nearly three times the risk of developing DCI compared to those with grades I-II (aOR 2.92, 95 % CI 1.83–4.66). The pathophysiological mechanism underlying this association involves the release of vasoactive substances from lysed erythrocytes, particularly oxyhemoglobin, which triggers endothelin-1 production and reduces nitric oxide bioavailability (Macdonald et al., 2007b). The resultant imbalance between vasoconstrictive and vasodilatory factors creates a microenvironment conducive to sustained arterial narrowing. Recent metabolomic studies have further identified specific breakdown products of hemoglobin that correlate with AVS severity, potentially offering new therapeutic targets (Leclerc et al., 2015).

Among the dynamic clinical parameters, pathological arterial blood pressure values, elevated body temperature, and restlessness emerged as significant early predictors of DCI development. Alterations in arterial blood pressure (aOR 1.76, 95 % CI 1.15–2.70) may reflect dysregulation of cerebral autoregulation mechanisms or represent compensatory responses to declining cerebral perfusion (Solenski et al., 1995). Pathological body temperature elevations (aOR 2.38, 95 % CI 1.54–3.67) likely indicate inflammatory processes that not only accompany but potentially exacerbate AVS and DCI through augmented cytokine release and endothelial dysfunction (Provencio and Vora, 2005).

Notably, restlessness (aOR 2.10, 95 % CI 1.36–3.24) deserves special attention as an early harbinger of DCI. This finding aligns with observations by Doerksen and Naimark (2006b), who identified nonspecific behavioral changes as early indicators of developing DCI. The neurobiological basis for this association may relate to subtle perfusion deficits affecting subcortical structures involved in arousal and emotional regulation before clinically detectable ischemia in eloquent cortical areas (Rabinstein et al., 2005).

The appearance of new neurological deficits (aOR 4.85, 95 % CI 3.04–7.75) and deterioration on the GCS by ≥ 2 points (aOR 6.33, 95 % CI 3.96–10.11) represented the strongest predictors of DCI. While these parameters inherently overlap with the definition of DCI itself (Vergouwen et al., 2010c), their inclusion in the predictive model is justified as they identify patients requiring urgent diagnostic evaluation and aggressive therapeutic intervention. We acknowledge that this overlap introduces circularity; even so, these changes often predate formal DCI diagnosis, making them clinically invaluable for early risk identification. In a similar fashion, variables such as fever, blood pressure instability, and restlessness are nonspecific but represent relevant bedside warning signs, despite potential confounding by infection or systemic illness. Moreover, these manifestations may occur on a spectrum of severity, with milder changes potentially indicating evolving DCI before reaching the threshold that satisfies formal diagnostic criteria.

### 4.2 The Delayed Cerebral Ischemia Scoring System (DCISS)

Based on the identified independent predictors, a novel scoring system, DCISS, that enables risk stratification for DCI development was developed. This system demonstrated excellent discriminative ability (AUC = .89, 95 % CI .86–.92) with high sensitivity (88.5 %) and specificity (82.0 %) at the optimal cut-off value of 8 points. The score's performance remained robust after internal validation using bootstrap resampling, with minimal evidence of overfitting.

The key advantage of the developed scoring system compared to existing models (Frontera et al., 2006; Kramer et al., 2008) is its incorporation of not only baseline clinical and radiological parameters (age, Fisher grade) but also dynamic clinical variables monitored throughout hospitalization (pathological arterial blood pressure, pathological body temperature, restlessness, new neurological deficit, deterioration of GCS). This approach permits continuous risk re-stratification during the high-risk period for DCI development (days 3–14 post-ictus), enabling clinicians to adjust therapeutic interventions in accordance with evolving clinical presentations.

The stratification of patients into four risk categories (low, moderate, high, and very high) based on DCISS scores provides clinically meaningful decision thresholds. Patients classified as low risk (DCISS 0–3) demonstrated a minimal 3.2 % incidence of DCI and excellent outcomes (87.2 % favorable outcome at discharge). In contrast, the very high-risk group (DCISS ≥14) exhibited an 82.5 % incidence of DCI with only 17.5 % achieving favorable outcomes at discharge. This pronounced risk gradient validates the scoring system's clinical utility and provides a framework for tailored management strategies.

Nevertheless, the DCISS should be regarded as an exploratory model rather than a definitive predictive tool. Due to the single-center design, modest sample size, and long (10-year) inclusion period, the developed score may reflect center-specific practices and temporal variations in management. Hence, the findings should be considered hypothesis-generating; the DCISS requires external (prospective) validation in larger, well-defined multicenter cohorts before clinical adoption. The DCISS offers several practical applications in the clinical management of aSAH patients. First, it facilitates early identification of high-risk patients who may benefit from intensified monitoring protocols, including more frequent neurological examinations, transcranial Doppler ultrasonography, and potentially prophylactic interventions. Second, it provides objective criteria for resource allocation decisions, including appropriate triage to high-dependency units and selection of patients who might benefit from advanced neuroimaging surveillance. Third, the scoring system could serve as a standardized risk assessment tool for clinical trials evaluating novel AVS and DCI prevention strategies, enabling more homogeneous cohort selection and enhancing statistical power.

Potential preventive interventions for high-risk patients (DCISS ≥8) include optimization of hemodynamic parameters through euvolemic therapy, careful blood pressure management, and aggressive fever control (Treggiari et al., 2003b). For patients in the very high-risk category (DCISS ≥14), consideration might be given to prophylactic endovascular interventions such as transluminal balloon angioplasty, which has shown promise in preventing DCI in selected cases (Jun et al., 2010). Additionally, such patients might benefit from emerging pharmacological therapies targeting the inflammatory cascade, including phosphodiesterase inhibitors, endothelin receptor antagonists, and free radical scavengers, which have demonstrated varying degrees of efficacy in experimental and early clinical studies (Hong et al., 2014).

### 4.3 Clinical implications and future directions

The relationship between DCISS scores and clinical outcomes was striking, with higher scores strongly associated with increased mortality, longer hospitalization, and worse functional outcomes at both discharge and 3-month follow-up. This association remained robust across all predefined risk categories, suggesting that the pathophysiological processes driving DCI exert lasting effects on neurological recovery. Moreover, the ability of the DCISS to predict not only DCI but also functional outcomes enhances its clinical relevance as a prognostic tool.

Recent advances in neuroimaging and biomarker research offer exciting opportunities to further refine DCI prediction. Perfusion imaging techniques, including CT perfusion and arterial spin labeling MRI, can detect subtle alterations in cerebral blood flow before clinical manifestations become apparent (Dhar et al., 2012). Additionally, emerging biomarkers such as elevated CSF lactate and glucose levels, matrix metalloproteinases, vascular endothelial growth factor, and specific microRNA profiles may complement clinical parameters in DCI prediction models (Messina et al., 2023; Wang et al., 2021). Integration of these advanced diagnostic modalities with clinical scoring systems like the DCISS could potentially enhance predictive accuracy and enable increasingly personalized management strategies.

Machine learning approaches represent another promising avenue for refining DCI prediction. By analyzing complex patterns within longitudinal clinical data, neuroimaging parameters, and biomarker profiles, artificial intelligence algorithms may identify subtle relationships that elude traditional statistical methods (Megjhani et al., 2021). The development of dynamic prediction models that continuously update risk assessments based on evolving clinical data could further enhance the precision of DCI prediction and facilitate timely intervention.

### 4.4 Limitations and strengths

Several limitations should be acknowledged when interpreting findings. First, as a single-center study, these results may reflect institution-specific practices and patient populations, potentially limiting generalizability. External validation in diverse clinical settings is warranted to confirm the broad applicability of the DCISS. Prospective studies in the future specifically designed to evaluate and refine the DCISS will be essential to establish its predictive power and clinical reliability. Second, the authors could not analyze certain potentially relevant biomarkers or advanced imaging parameters that might enhance predictive accuracy. Third, while the authors performed internal validation using bootstrapping, external validation in an independent cohort remains essential to definitively establish the scoring system's predictive value. Fourth, the stepwise logistic regression and dichotomized continuous variables (e.g., age <55 years) may have modestly reduced model robustness. This warrants future refinement using continuous modeling and modern validation techniques.

Despite these limitations, this study possesses several notable strengths. The large sample size (n = 524) provided robust statistical power for multivariate analysis and model development. The comprehensive assessment of both baseline and dynamic clinical parameters captured the multifaceted nature of DCI pathophysiology. The rigorous methodological approach, including bootstrap validation and decision curve analysis, enhanced the reliability of this study's findings. Finally, the development of a straightforward, clinically applicable scoring system increases the translational potential of our research.

## 5 Conclusions

This retrospective analysis of aSAH highlights vasospasm as a near-universal radiological finding, with DCI affecting 30 % of patients. The developed the Delayed Cerebral Ischemia Scoring System (DCISS), integrated baseline factors (age, WFNS/Fisher grades) and dynamic clinical markers (pathological blood pressure, fever, restlessness, neurological deterioration) to stratify risk (AUC = .89). The DCISS represents a preliminary, internally validated tool that may assist in targeted monitoring and risk stratification; further prospective validation is required before routine clinical implementation. Its strong correlation with poor functional outcomes (discharge and 3-month follow-up) underscores the need for early intervention in high-risk patients. External validation, integration of novel biomarkers, and AI-driven refinement are critical next steps. By optimizing risk prediction and personalized management, the DCISS has the potential to reduce morbidity and mortality from this devastating complication.

## Consent to participate

All participants or next-of-kin have provided written informed consent to participate in this study.

## Consent to publish

N/A (no identifable patient data/imaging included).

## Ethics approval

The study was approved by the Institutional Review Board (IRB) of the University Clinical Center of Vojvodina (Ethics approval number: 00–20/118-2015).

## Author contributions

DD, DN, and MBN contributed to the study conception and design. Material preparation and analysis were performed by DD and DN. Data collection was performed by NL and DD. The first draft of the manuscript was written by DD and DN, and all authors commented on previous versions of the manuscript. All authors read and approved the final manuscript.

## Funding

This research received no funding.

## Conflict of interest

The authors declare that they have no known competing financial interests or personal relationships that could have appeared to influence the work reported in this paper.

## Data Availability

The data that support the findings of this study are available from the corresponding author upon reasonable request.
