# Supplementary material for: Predictive model and scoring system for delayed cerebral ischemia following aneurysmal subarachnoid hemorrhage: A ten-year prospective analysis of observational data
Source: Brain Spine. 2025 Nov 19;5:105885. doi: 10.1016/j.bas.2025.105885 (PMC12681525; doi:10.1016/j.bas.2025.105885)
Supplement: Multimedia component 1 [file mmc1.docx]

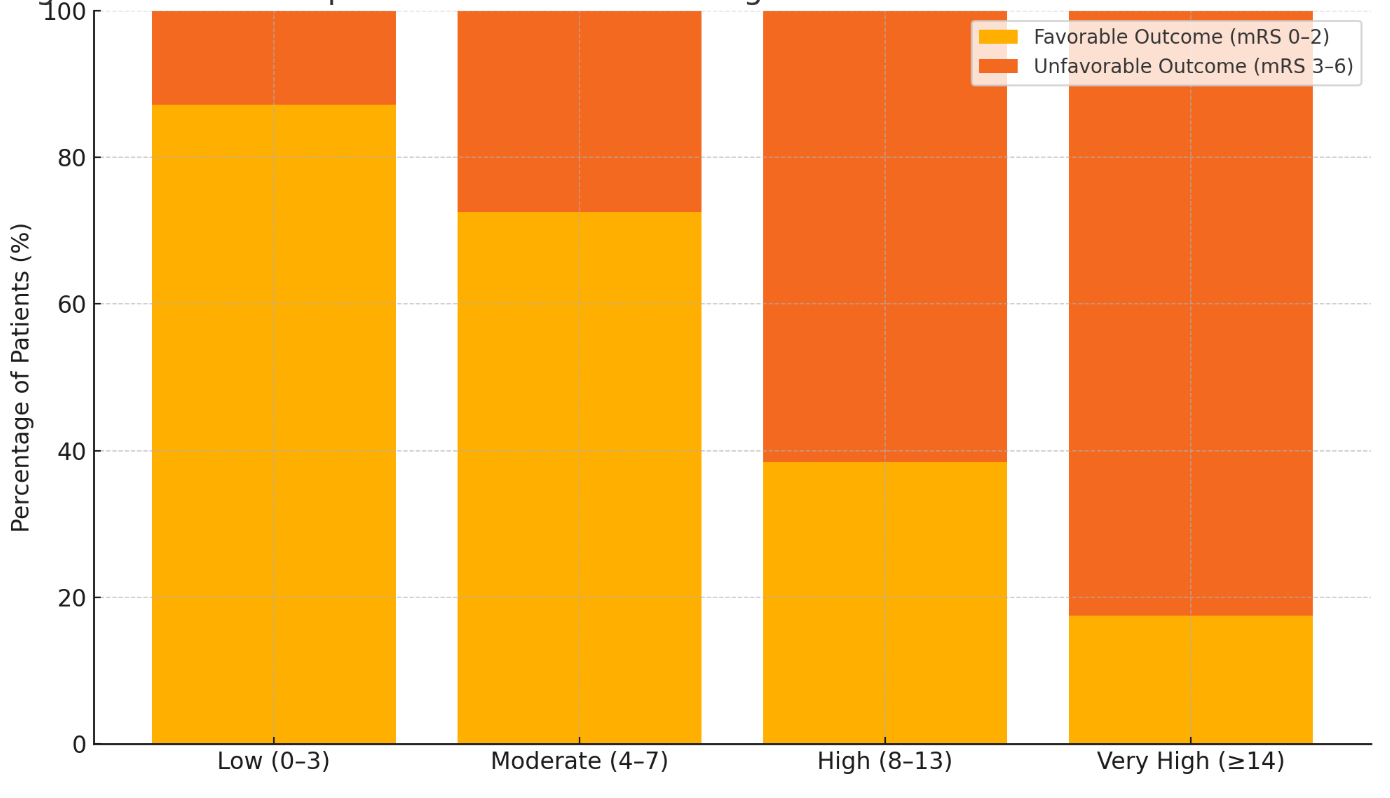


**Fig. S1. Relationship Between** **DCISS Risk Categories and Functional Outcomes at Discharge.** This figure displays the distribution of functional outcomes at discharge across four DCISS risk categories: low (0–3 points), moderate (4–7 points), high (8–13 points), and very high (≥14 points). Outcomes are stratified as favorable (modified Rankin Scale [mRS] 0–2) and unfavorable (mRS 3–6). A clear inverse relationship is observed between DCISS score and likelihood of favorable outcome: patients in the low-risk category achieved favorable outcomes in 87.2% of cases, while only 17.5% of very high-risk patients had favorable outcomes. This highlights the prognostic value of the DCISS in predicting post-hemorrhage functional recovery. Among patients with low DCISS scores (0-3), 87.2% had favorable outcomes, compared to 72.5% with moderate scores (4-7), 38.4% with high scores (8-13), and only 17.5% with very high scores (≥14) (p < .001).


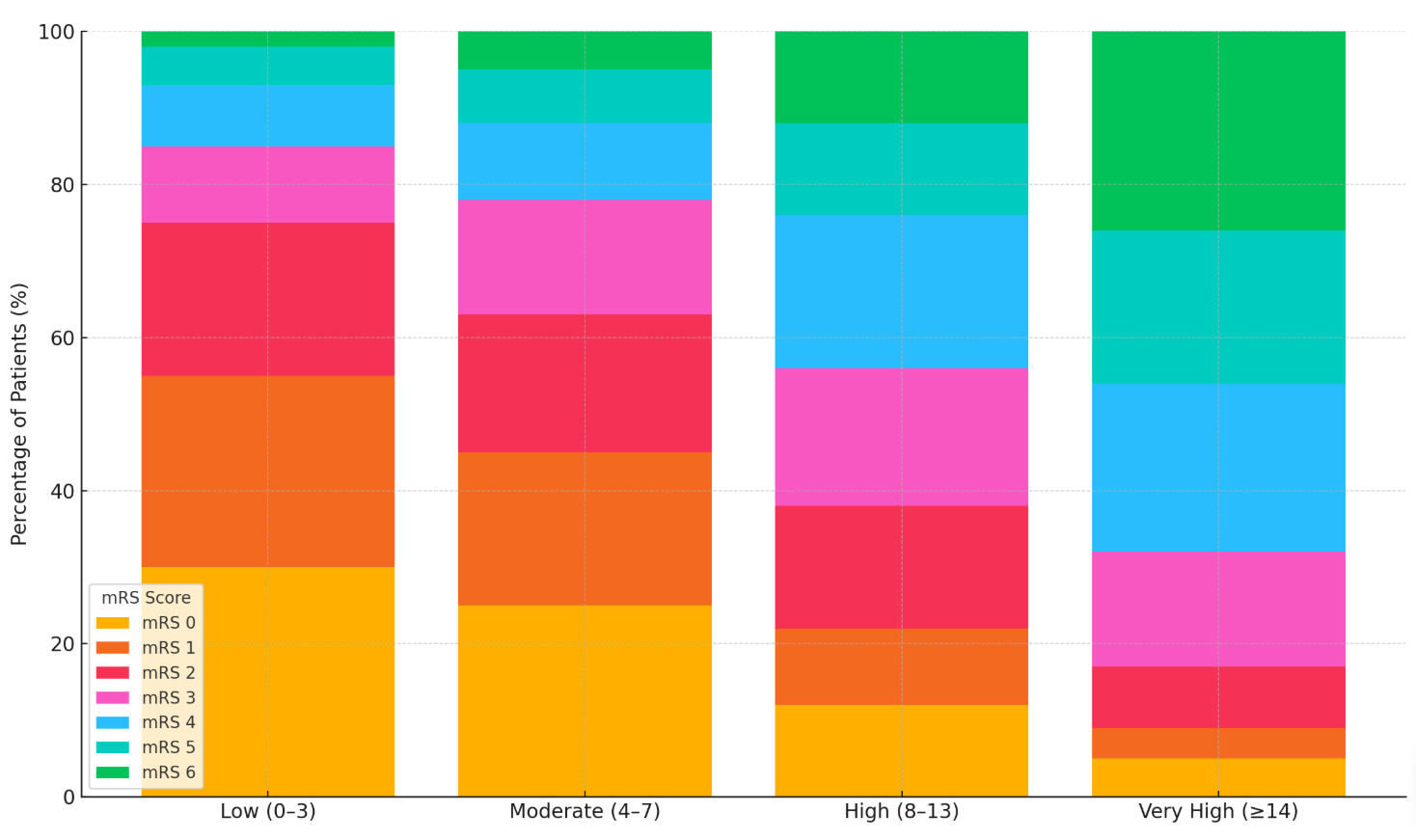


**Fig. S2 Distribution of mRS Scores at 3-Month Follow-up According to DCISS Risk Categories.** This figure presents the detailed distribution of functional outcomes, assessed by the modified Rankin Scale (mRS), at 3 months post-aSAH, stratified by DCISS-defined risk categories. Patients were classified into low (0–3 points), moderate (4–7 points), high (8–13 points), and very high (≥14 points) risk groups. A clear shift towards worse functional outcomes (higher mRS scores) is observed as the DCISS risk category increases. While most low-risk patients achieved excellent functional recovery (mRS 0–2), very high-risk patients exhibited a significantly greater proportion of severe disability (mRS 4–5) and mortality (mRS 6). These findings underscore the strong prognostic value of the DCISS not only in predicting DCI but also in anticipating long-term functional outcomes.
