# Supplementary material for: Predictive model and scoring system for delayed cerebral ischemia following aneurysmal subarachnoid hemorrhage: A ten-year prospective analysis of observational data
Source: Brain Spine. 2025 Nov 19;5:105885. doi: 10.1016/j.bas.2025.105885 (PMC12681525; doi:10.1016/j.bas.2025.105885)
Supplement: Multimedia component 2 [file mmc2.docx]

| Cut-off Value | Sensitivity (% , 95% CI) | Specificity (% , 95% CI) | PPV (% , 95% CI) | NPV (% , 95% CI) | Accuracy (% , 95% CI) | Youden Index |
| --- | --- | --- | --- | --- | --- | --- |
| ≥4 | 97.5 (93.7–99.3) | 38.7 (33.7–43.9) | 38.8 (33.9–43.8) | 97.4 (93.5–99.3) | 56.1 (51.8–60.4) | 0.362 |
| ≥5 | 96.2 (91.8–98.6) | 49.6 (44.4–54.8) | 43.8 (38.9–49.2) | 97.1 (91.7–99.0) | 63.2 (59.0–67.2) | 0.458 |
| ≥6 | 94.3 (89.4–97.4) | 61.3 (56.1–66.3) | 49.2 (43.5–54.9) | 96.2 (93.4–98.4) | 71.2 (67.1–75.0) | 0.556 |
| ≥7 | 92.4 (87.0–96.0) | 70.8 (65.9–75.4) | 55.6 (49.5–61.5) | 95.9 (92.9–97.9) | 77.3 (73.5–80.8) | 0.632 |
| **≥8*** | **88.5 (82.5–93.1)** | **82.0 (77.7–85.8)** | **67.1 (60.3–73.3)** | **94.7 (91.6–96.9)** | **84.0 (80.5–87.0)** | **0.705**** |
| ≥9 | 79.6 (72.5–85.6) | 88.3 (84.5–91.4) | 74.7 (67.5–81.0) | 90.8 (87.2–93.7) | 85.7 (82.3–88.6) | 0.679 |
| ≥10 | 72.0 (64.3–78.8) | 91.6 (88.2–94.2) | 78.9 (71.3–85.2) | 88.3 (84.6–91.4) | 85.7 (82.3–88.6) | 0.636 |
| ≥11 | 63.1 (55.0–70.6) | 94.0 (91.1–96.2) | 81.7 (73.5–88.3) | 85.3 (81.6–88.7) | 84.5 (81.1–87.6) | 0.571 |
| ≥12 | 51.0 (42.9–59.0) | 96.2 (93.7–97.9) | 84.2 (74.7–91.2) | 83.0 (78.2–86.4) | 83.2 (79.7–86.3) | 0.472 |
| ≥14 | 33.1 (25.9–41.0) | 98.4 (96.5–99.4) | 90.7 (79.5–96.8) | 78.1 (74.3–81.6) | 79.0 (75.2–82.5) | 0.315 |
| ≥16 | 19.7 (13.9–26.8) | 99.5 (97.9–99.9) | 93.9 (79.8–98.3) | 75.1 (71.8–78.7) | 75.8 (71.9–79.3) | 0.192 |
| **Abbreviations:** CI = confidence interval; PPV = positive predictive value; NPV = negative predictive value.  * Optimal cut-off value determined by the Youden index (sensitivity + specificity - 1).  ** Maximum Youden index value. | | | | | | |

**Table S1.** Performance Characteristics of the DCISS at Various Cut-off Points

**Table S2.** Causes of Death in Patients with aSAH

| **Cause of Death** | **Total (n=68)** | **DCI (n=39)** | **Non-DCI (n=29)** | **p-value** |
| --- | --- | --- | --- | --- |
| DCI-related cerebral infarction, n (%) | 27 (39.7) | 27 (69.2) | 0 (0.0) | < .001* |
| Direct effects of initial hemorrhage, n (%) | 20 (29.4) | 4 (10.3) | 16 (55.2) | < .001* |
| Rebleeding, n (%) | 9 (13.2) | 3 (7.7) | 6 (20.7) | .120 |
| Procedure-related complications, n (%) | 5 (7.4) | 1 (2.6) | 4 (13.8) | .082 |
| Hydrocephalus, n (%) | 3 (4.4) | 1 (2.6) | 2 (6.9) | .379 |
| Medical complications, n (%) | 4 (5.9) | 3 (7.7) | 1 (3.4) | .462 |
| Timing of Death |  |  |  |  |
| Very early (≤3 days), n (%) | 21 (30.9) | 2 (5.1) | 19 (65.5) | < .001* |
| Early (4–14 days), n (%) | 35 (51.5) | 28 (71.8) | 7 (24.1) | < .001* |
| Late (>14 days), n (%) | 12 (17.6) | 9 (23.1) | 3 (10.3) | .167 |
| DCISS Risk Categories |  |  |  |  |
| Low risk (0–3), n (%) | 4 (5.9) | 1 (2.6) | 3 (10.3) | .173 |
| Moderate risk (4–7), n (%) | 12 (17.6) | 4 (10.3) | 8 (27.6) | .063 |
| High risk (8–13), n (%) | 23 (33.8) | 12 (30.8) | 11 (37.9) | .541 |
| Very high risk (≥14), n (%) | 29 (42.6) | 22 (56.4) | 7 (24.1) | .008* |
| Median DCISS score (IQR) | 12 (8–16) | 14 (10–18) | 8 (5–12) | < .001* |

**Abbreviations:** DCISS = Delayed Cerebral Ischemia Scoring System; IQR = interquartile range.
**Note:** Medical complications included pneumonia (n = 2), sepsis (n = 1), and pulmonary embolism (n =1).
*Statistically significant (p < .05).
